# Vitamin D-Related Single Nucleotide Polymorphisms as Risk Biomarker of Cardiovascular Disease

**DOI:** 10.3390/ijms23158686

**Published:** 2022-08-04

**Authors:** Paula González Rojo, Cristina Pérez Ramírez, José María Gálvez Navas, Laura Elena Pineda Lancheros, Susana Rojo Tolosa, María del Carmen Ramírez Tortosa, Alberto Jiménez Morales

**Affiliations:** 1 Pharmacogenetics Unit, Pharmacy Service, University Hospital Virgen de las Nieves, 18014 Granada, Spain; 2 Department of Biochemistry and Molecular Biology II, Faculty of Pharmacy, Campus Universitario de Cartuja, University of Granada, 18071 Granada, Spain

**Keywords:** cardiovascular disease, risk, *VDR*, polymorphisms, biomarkers

## Abstract

Cardiovascular diseases (CVDs) are a group of disorders of the heart and blood vessels. In addition to environmental risk factors, genetic predisposition increases the risk; this includes alterations in the vitamin D receptor gene (*VDR*). These alterations play a key role in modifying vitamin D uptake, being able to modify its function and increasing susceptibility to cardiovascular disorders. The aim of this study was to evaluate the association of polymorphisms in the *VDR* gene and risk of CVD in a Caucasian population. A retrospective case-control study was conducted comprising 246 CVD patients and 246 controls of Caucasian origin from Southern Spain. The genetic polymorphisms *BsmI* (rs1544410), *TaqI* (rs731236), *ApaI* (rs7975232), *FokI* (rs2228570) and *Cdx2* (rs11568820) were determined by means of real-time polymerase chain reaction (PCR) for allelic discrimination using TaqMan^®^ probes. The logistic regression analysis adjusted for body mass index and diabetes revealed that the TT genotype was associated with a higher risk of CVD in both the genotypic model (*p* = 0.0430; OR = 2.30; 95% CI = 1.06–5.37; TT vs. CC) and the recessive model (*p* = 0.0099; OR = 2.71; 95% CI = 1.31–6.07; TT vs. C). Haplotype analysis revealed that the haplotype GAC (*p* = 0.047; OR = 0.34; 95% CI = 0.12–0.98) was associated with increased risk of CVD. The *VDR* polymorphisms *FokI* (rs2228570) was significantly associated with the development of CVD. No influence was observed of the *VDR* polymorphisms *BsmI* (rs1544410), *TaqI* (rs731236), *ApaI* (rs7975232) and *Cdx2* (rs11568820) on the risk of developing CVD in the patients studied.

## 1. Introduction

Cardiovascular diseases (CVDs) are a group of disorders of the heart and blood vessels. They are classified into coronary heart disease, cerebrovascular disease, peripheral arteriopathies, rheumatic heart disease, congenital heart disease, deep vein thrombosis, and pulmonary embolism. According to the World Health Organization, CVD is the leading cause of death worldwide. It is estimated that in 2015 (the last year for which data have been published) 17.7 million people died from CVDs, representing 31% of all registered global deaths. Of these deaths, more than 80% take place in low- and middle-income countries, affecting men and women almost equally [1]; hence the utmost importance of identifying the risk factors involved in the development of this disease, given the enormous social and economic implications [2].

Although the etiology of CVD has not been clearly established, it has been shown that the causes of these disorders are multi-factorial, due to the combination of environmental risk factors, such as smoking, lack of physical activity, eating habits, high blood pressure, type 2 diabetes, and dyslipidemias, with genetic predisposition [3,4,5]. The search for genes that predispose to CVD has led to the identification of human variations of deoxyribonucleic acid (DNA), evaluation of the risk profile and adoption of preventive or therapeutic measures [4]. The vitamin D receptor (*VDR*) gene and its single nucleotide polymorphisms (SNPs) have received special attention due to their association with a cardiometabolic risk profile. The precise mechanism underlying its influence on pathogenesis is still unclear and may be the result of different factors [6,7,8]. Firstly, the *VDR* gene is found in vascular smooth muscle cells and endothelial cells, potentially affecting their growth and proliferation [9]. In addition, *VDR* activation induces an increase dependent on nitric oxide concentration in endothelial cells, and enhances the angiogenic properties of endothelial progenitor cells [10,11]. Secondly, vitamin D could regulate immune cells, inhibiting the release of pro-inflammatory cytokines and increasing the release of anti-inflammatory cytokines, thus playing a role in blood vessel protection [12]. Thirdly, vitamin D is an important regulator of the renin-angiotensin-system (RAS). Vitamin D deficiency would involve activation of the renin gene, producing an increase in angiotensin II, which can lead to hypertension and ventricular hypertrophy [13,14]. Additionally, it would prompt an increase in the production of reactive oxygen species (ROS) and the activation of G proteins such as Rho A, resulting in the inhibition of the pathways necessary for intracellular glucose transport and, therefore, the development of insulin resistance and the onset of metabolic syndrome [15]. Finally, vitamin D is associated with an atherogenic lipid profile that includes increased serum LDL and decreased HDL levels. The biologically active form of vitamin D suppresses foam cell formation, decreases cholesterol uptake by macrophages, and induces LDL autophagy through gene regulation through *VDR*. However, short-term replenishment of 25-hydroxyvitamin D levels does not improve the lipid profile in humans [16,17]. In short, vitamin D can lower blood pressure values and have anti-inflammatory, anti-proliferative, anti-hypertrophic, anti-fibrotic, anti-diabetic, and anti-thrombotic effects, beneficially modulating classic cardiovascular risk factors [18].

Vitamin D can be obtained from 7-dehydrocholesterol after exposure to sunlight and through food [19,20,21]. Vitamin D is hydroxylated to 25-hydroxyvitamin D_3_ (25(OH)D_3_) in the liver and 1α-hydroxylated in the kidney, forming the active hormone 1,25-dihydroxyvitamin D_3_ (1.25(OH)_2_D_3_) [22,23]. This active metabolite binds to the *VDR* ligand-binding domain (LBD), forming a heterodimer with the retinoid X receptor (RXR) that binds to vitamin D response elements (VDRE) in the promoter region of target genes modulating transcriptional activation [24,25]. The *VDR* is expressed almost ubiquitously regulating approximately 3% of the genome; more than 900 genes participate in many physiological processes [26,27].

The gene encoding the nuclear vitamin D receptor is a large gene more than 100 Kb in length [28], found in the long arm of chromosome 12 (locus 12q13.1) [29]. It has at least five promoter regions, eight coding exons and at least six non-coding exons that are alternately spliced [30]. It encodes a protein that contains 427 amino acids [26] belonging to the family of steroid receptors for retinoic acid, thyroid hormone, sex hormones, and adrenal steroids [31]. To date, 470 SNPs have been identified at the *VDR* locus [32], but there are five common polymorphisms that have been widely studied due to their effects on various physiological and pathological phenotypes; *Cdx2* (rs11568820), *FokI* (rs2228570), *TaqI* (rs731236), *BsmI* (rs1544410) and *ApaI* (rs7975232) [33,34].

Recently, the importance of these polymorphisms and their haplotypes has been increasingly recognized as more studies have linked them to different diseases [35]. Some SNPs play a key role in modifying 1.25(OH)_2_D_3_ uptake, due to their capacity to modify vitamin D function [36]. However, the exact molecular mechanism explaining the association between *VDR* polymorphisms and serum levels of 25(OH)_2_D_3_ remains unknown [37]. The *VDR* SNP *FokI* (rs2228570, exon 2, C > T, formerly known as rs10735810) is characterized by the presence of two ATG start codons separated by six nucleotides, modifying the length and functional activity of the protein. Thus, *FokI* is the only *VDR* polymorphism with functional impact, as it involves the loss of a transcription start site. It is not in linkage disequilibrium with other SNPs, so the associations with the *VDR* genotype *FokI* are considered independent markers of the *VDR* gene [29]. The C allele results in a shorter variant of the *VDR* protein (424 amino acids) and a longer variant associated with the T allele (427 amino acids) [38]. Although no significant differences have been reported in ligand affinity, DNA binding or transactivation activity between the two allelic forms of *FokI*, the shorter variant shows greater receptor activity than the longer variant as it appears to interact more efficiently with the transcription factor TFIIB [39,40]. The SNPs *BsmI* (rs1544410, intron 8, G > A), *TaqI* (rs731236, exon 9, T > C) and *ApaI* (rs7975232, intron 8, C > A) are found near the 3’UTR region of the *VDR* gene [6,27,35]. This region contains many polymorphisms and therefore a strong linkage disequilibrium could explain the associations observed with *Bsm-Apa-Taq*. These polymorphisms are involved in the regulation of VDR expression, notably through regulation of messenger ribonucleic acid (mRNA) stability [41,42]. Therefore, they can influence the expression of the protein without altering its structure or function [43]. The polymorphism *Cdx2* (rs11568820, exon 1, G > A) is located in the promoter region of the 5’ end of the *VDR* gene. *The VDR* specifically interacts with functional enhancer elements in the *VDR* gene in the small intestine, regulating its gene expression and therefore calcium absorption [44,45]. The transcriptional activity of the *VDR* promoter with the G allele is lower compared to the A allele, decreasing intestinal absorption of calcium [44] and potentially influencing central obesity [27].

Numerous studies have been carried out under this conceptual framework to investigate the association of these polymorphisms with the risk of developing CVD. However, the results obtained are contradictory and further studies are required in different populations to obtain more information about the influence of these SNPs on the susceptibility of developing CVD. Based on the foregoing, we carried out this study to evaluate the association of these polymorphisms in the *VDR* gene (*BsmI* (rs1544410), *TaqI* (rs731236), *ApaI* (rs7975232), *FokI* (rs2228570) and *Cdx2* (rs11568820)) and CVD risk in a Caucasian population.

## 2. Results

### 2.1. Patients Characteristics

A total of 246 cases with CVD and 246 controls of Caucasian origin from Southern Spain were included in the study. Controls were matched to cases on age and sex (1:1). Their clinical, socio-demographic, and pathological characteristics are detailed in Table 1. The median age of the patients was 70 (63.25, 76) years. The group of cases consisted of 132 women (53.7%) and 114 men (46.3%). Most of the cases were non-smokers, non-drinkers, overweight, not dyslipidemic, hypertensive and did not have diabetes. As regards cardiovascular disease, 84 (34.14%) suffered from cardiac arrhythmias, 15 (6.10%) presented cardiomyopathy, 15 (6.10%) cerebrovascular disease, 27 (10.98%) heart failure, 46 (18.70%) valvular heart disease and 59 (23.98%) vascular peripheral disease.

The control group consisted of 142 women (142/246; 57.7%) and 104 men (104/246; 42.3%), with a mean age of 69.5 (63, 76) years. Most of the controls were non-smokers, non-drinkers, had a healthy weight, not dyslipidemic, did not have hypertensive and did not have diabetes.

Cases and controls showed significant differences in terms of BMI (*p* = 0.0003) and diabetes (*p* < 0.001; OR = 10.53; 95% CI = 1.32–3.03; diabetes vs. non-diabetes).

### 2.2. Genotype Distribution

**Figure 1:**
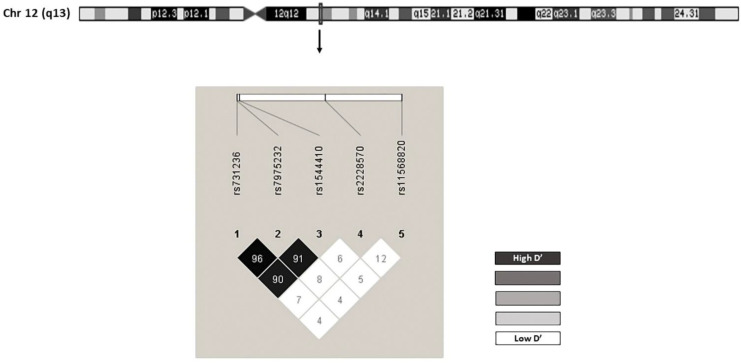
Chromosomal location for (vitamin D receptor) *VDR*, and linkage disequilibrium (LD).

The genotype frequency for the control group coincided with the expected values according to the Hardy-Weinberg equilibrium model (Table S1). The coefficients D’ and r2 for the evaluation of linkage disequilibrium are described in Table S2 and Figure 1. All SNPs had minor allele frequencies above 1%, so none of them had to be excluded from the analysis (Table S3). The estimated haplotype frequencies are presented in Table S4.

### 2.3. Influence of Genetic Polymorphisms on CVD Risk

Bivariate analysis was performed according to the following models: genotypic, additive, allelic, dominant, and recessive. *VDR FokI* (rs2228570) was the only polymorphism to show an association with CVD risk in the genotypic (*p*_χ²_ = 0.0063), additive (*p*_χ²_ = 0.0267), allelic (*p*_χ²_ = 0.0230) and recessive (*p*_χ²_ = 0.0015) models (Table S5). This significant association was also found after making adjustments for the Bonferroni test, in which both the genotypic and recessive models showed that patients with the TT genotype presented a higher risk of developing CVD (p_Bonferroni-corrected_ = 0.0305; OR = 2.35; 95% CI = 1.33–4.28; TT vs. CC and p_Bonferroni-corrected_ =0.0075; OR = 2.39; 95% CI = 1.39–4.24; TT vs. C; Table 2, respectively). The logistic regression analysis adjusted for BMI and diabetes revealed that the TT genotype was associated with a higher risk of CVD in both the genotypic model (*p* = 0.0430; OR = 2.30; 95% CI = 1.06–5.37; TT vs. CC; Table 3) and the recessive model (*p* = 0.0099; OR = 2.71; 95% CI = 1.31–6.07; TT vs. C; Table 3). However, no association was found in either the genotypic logistic regression model adjusted for BMI and diabetes (*p* = 0.2286; OR = 0.72; 95% CI = 0.43–1.22; TC vs. CC; Table 3), the dominant model (*p* = 0.8648 OR = 0.95; 95% CI = 0.58–157; T vs. CC; Table 3) or the additive model (*p* = 0.2151; OR = 1.24; 95% CI = 0.88–1.76; T vs. C; Table 3). The other polymorphisms analyzed did not show statistically significant associations with the development of CVD in any of the models studied (Table S5). Haplotype analysis revealed that the GAC haplotype (*p* = 0.047; OR = 0.34; 95% CI = 0.12–0.98) was associated with an increased risk of CVD (Table 4).

## 3. Discussion

Cardiovascular disease, which involves the heart, brain, and peripheral circulation, represents a major health problem throughout the world. It has multiple genetic and environmental components that contribute to the observed phenotype [46]. The role played by vitamin D in cardiovascular function is receiving more and more attention and, in particular, the effect of the respective polymorphisms in the *VDR* gene. Most studies describing *VDR* SNPs have focused on five polymorphisms: *BsmI* (rs1544410), *TaqI* (rs731236), *ApaI* (rs7975232), *FokI* (rs2228570) and *Cdx2* (rs11568820) [32,33].

The *VDR FokI* polymorphism (rs2228750) is one of the most researched. It is the only *VDR* polymorphism with functional impact, as it involves the loss of a transcription start site [29]. Previous studies have reported contradictory results, so the relationship between this polymorphism and the development of CVD is not clear [47,48,49,50,51,52,53,54]. In our study, the logistic regression analysis adjusted for body mass index and diabetes revealed that the *VDR FokI* TT genotype showed an association with increased risk of CVD in the genotypic model (*p* < 0.05; Table 3) and in the recessive model (*p* < 0.05; Table 3). Several studies are consistent with our results, for example one study conducted in a Caucasian population (from Poland) (58 cases/142 controls), in which statistically significant differences were found in relation to the T allele of the *VDR FokI* polymorphism, identifying it as a risk factor for heart failure and hypertension (*p* = 0.03) [55]. Another study in a Caucasian population (from Egypt) (50 cases/50 controls) also reported an association between the *VDR FokI* polymorphism and the presence of congenital heart disease defects. In particular, patients carrying the *VDR FokI-*T allele were at greater risk of suffering congenital heart disease defects [(*p* = 0.006; OR = 3; 95% CI = 1–8; TC vs. CC); (*p* = 0.03; OR = 10; 95% CI = 1–97; TT vs. CC); (*p* = 0.001; OR = 3; 95% CI = 2–6; T vs. C)] [50]. Another study in a Caucasian population (from Egypt) (244 cases/138 controls) shows that allele T frequency is higher in patients with CVD (group A and B) which means *VDR FokI*-T allele increase CVD risk [(*p* < 0.001; OR = 2.397; 95% CI = 1.518–3.786; C vs. T); (*p* < 0.001; OR = 2.298; 95% CI = 1.443–3.660; C vs. T) [56]. Furthermore, the study carried out by Nakhl et al. (2019) in a Caucasian population (Mediterranean region) with 50 patients reported that the presence of *VDR FokI* polymorphisms is higher compared to the rest of the Caucasian population [46]. Moreover, the results of our study are in accord with others conducted in an Asian population. For example, the study of Hao et al. (2019) in a Chinese population (145 cases/90 controls), in which statistically significant differences were found between cases and controls in relation to the T allele of the *VDR FokI* polymorphism, identifying it as a risk factor for the disease [(*p* < 0.0001; OR = 2.45; 95% CI = 1.60–3.77; T vs. C); (*p* < 0.0001; OR = 3.42; 95% CI = 1.94–6.07; TC vs. CC); (*p* = 0.02; OR = 4.73; 95% CI = 1.35–16.27; TT vs. CC)] [47]. Likewise, another study carried out in an Asian population (from China) (860 cases/862 controls) showed that individuals carrying the VDR *FokI-*T allele were at a higher risk of developing coronary artery disease (CAD) [(*p* < 0.001; OR = 1.47; 95% CI = 1.22–1.85; CT vs. CC); (*p* < 0.001; OR = 2.12; 95% CI = 1.43–2.88; TT vs. CC); (*p* < 0.001; OR = 1.68; 95% CI = 1.26–2.17; TC + TT vs. CC)] [48]. Similarly, a study carried out in an Asian population (from India) (292 cases/219 controls) reported a statistically significant association between the T genotype of the VDR *FokI* polymorphism and susceptibility to developing ischemic cerebrovascular accident (CVA) (*p* = 0.01; OR = 1.89; 95% CI = 1.17–3.07; TT vs. CC) [49]. Furthermore, according to the meta-analysis conducted by Tabaei et al. (2021) in Caucasian and Asian populations (3741 cases/1998 controls), the T allele of the VDR *FokI* SNP was significantly associated with the CAD risk in all genetic models including; dominant model (*p* = 0.02; OR = 1.47, 95% CI 1.06–2.03), recessive model (*p* < 0.001; OR = 1.39, 95% CI 1.14–1.71), allelic model (*p* = 0.01; OR = 1.38, 95% CI 1.08–1.76, *p* = 0.01), TT vs. CC model (*p* = 0.02; OR = 1.58, 95% CI 1.07–2.34), and TC vs. TT model (*p* = 0.04; OR = 1.38, 95% CI 1.00–1.92), having a strong significant association for the Asian population but not the Caucasian [57].

However, a study carried out in a Caucasian population (from Turkey) (54 cases/58 controls) evaluated the influence of the *VDR FokI* polymorphism on the risk of arteriovenous fistula (AVF), but found no statistically significant association (*p* = 0.168; OR = 1.683; 95% CI = 0.803–3.531) [52]. Another study carried out in a Caucasian population (United States) (205 cases/206 controls) also evaluated the influence of the *VDR FokI* polymorphism on the risk of congestive heart failure (HF) in patients with hypertension, without finding any statistically significant association in any of the analyzed models [(*p* > 0.05 OR = 0.82; 95% CI = 0.47–1.42; TC vs. CC); (*p* > 0.05; OR = 0.85; 95% CI = 0.39–1.88; TT vs. CC); (*p* > 0.05; OR = 0.82; 95% CI = 0.49–1.39; CT + TT vs. CC)] [53]. Finally, a meta-analysis composed of six studies in Caucasian and Asian populations (2725 cases/1017 controls) evaluated the influence of the *VDR FokI* polymorphism on the risk of CAD, but did not observe any statistically significant association in any of the models analyzed [(*p* > 0.05; OR = 0.93; 95% CI = 0.76–1.10; CT + TT vs. CC); (*p*> 0.05; OR = 1.14; 95% CI = 0.86–1.43; TT vs. CT + CC); (*p* > 0.05; OR = 1.00; 95% CI = 0.88–1.12; T vs. C); (*p* > 0.05; OR = 0.88; 95% CI = 0.71–1.06; CT vs. CC); (*p* > 0.05; OR = 1.06; 95% CI = 0.77–1.35; TT vs. CC)] [54].

According to the results shown in the majority of the studies, including ours, the *VDR FokI SNP* presents statistical association with CVD in Asian and Caucasian (from Europe) populations. However, this statistical significance does not occur in US Caucasian populations. It seems to indicate that ethnicity may influences this correlation.

Otherwise, *BsmI* (rs1544410), *TaqI* (rs731236) and *ApaI* (rs7975232) do not result in a structural change of the *VDR* protein. However, these polymorphisms can modify the expression of the *VDR* gene and increase susceptibility to developing CVD [29,43]. In our study, the *VDR BsmI* polymorphism did not show an association with susceptibility to developing CVD in any of the models analyzed (Table 2). Various studies in Caucasian populations are consistent with our results, such as the studies conducted by Ewida et al. (2021) [58] and Nakhl et al. (2019), in which there were no association between those polymorphisms and CVD [46]. However, according to the results of the study carried out by Abouzid et al. (2021) they suggest that *VDR BsmI*-AA genotype is protective of CVD (*p* = 0.04) [53]. Moreover, according to the meta-analysis by Tabaei et al. (2021) in Caucasian and Asian populations (6169 cases/2392 controls), *VDR ApaI-CC* genotype was significantly associated with CAD risk in all genetic models, including: dominant model (*p* = 0.002; OR = 1.20, 95% CI 1.07–1.34), allelic model (*p* = 0.004; OR = 1.12, 95% CI 1.04–1.21), CC vs. AA model (*p* = 0.01; OR = 1.22, 95% CI 1.04–1.44), and AC vs. AA model (*p* = 0.007; OR = 1.18, 95% CI 1.05–1.33), other than the recessive model (*p* = 0.17; OR = 1.10, 95% CI 0.96–1.27) [57].

Finally, the *Cdx2* polymorphism (rs11568820) is located in the promoter region of the 5′ end of the *VDR* gene regulating gene expression [44]. So far, no study has evaluated the impact of the *VDR Cdx2* polymorphism on the susceptibility for developing CVD. In our study, no significant association was found between the *VDR Cdx2* polymorphism (rs11568820) and the risk of developing CVD in any of the models analyzed (Table 2).

The main limitation of this study was the limited sample size compared to other studies, especially in terms of cases, which could have prevented the detection of certain associations. Another limitation of our study is that serum levels of 25(OH)D are not available as it is a retrospective study. Thus, it has not been possible to correlate the levels of active vitamin D and the genotypes. However, despite the limited sample size, after adjusting for the Bonferroni test to avoid false-positive associations, the effect of *VDR FokI* remained. The strengths of our study were the homogeneity of the sample, especially in terms of the cases, which consisted of only University Hospital Virgen de las Nieves patients diagnosed by the same team, and also from the same geographical area, thus increasing uniformity. In addition, the controls chosen were older than the cases to reduce potential selection bias.

## 4. Materials and Methods

### 4.1. Study Design

A retrospective case-control study was carried out.

### 4.2. Study Subjects

This included 246 patients with CVD and 246 controls of Caucasian origin from Southern Spain, with a case/control ratio 1. The cases were recruited at the University Hospital Virgen de las Nieves, Granada, Spain, from March 2013 to October 2022. Controls were individuals over 18 years of age who had resided in the same geographic area, with no personal history of CVD, recruited at the same hospital. This sample size has been chosen based on previous studies and the prevalence of these gene polymorphisms in a Spanish Caucasian population.

This case-control study was approved by the Ethics Committee of the Andalusian Health System (SAS) and carried out in accordance with the Declaration of Helsinki (code: 0957-N-21). All subjects participating in the study signed a written informed consent for the extraction of saliva or blood samples and their donation to the biobank. The samples were coded and treated confidentially.

### 4.3. Socio-Demographic and Clinical Variables

The socio-demographic data collected and included in the study were sex, age, smoking, alcoholic habit, body mass index (BMI), dyslipidemia, hypertension, diabetes, and cardiovascular disease suffered by the patients. Individuals were classified as non-smokers if they had never smoked or smoked < 100 cigarettes in their life, as ex-smokers if they had smoked ≥ 100 cigarettes in their life but were not currently smoking, and as active smokers if they had smoked ≥ 100 cigarettes in their life and were currently smoking. The individuals were classified according to standard drinking units (SDU) as non-drinkers if they were abstainers or did not consume alcohol regularly, as active drinkers if their alcohol consumption >4 SDU/day in men and >2.5 SDU/day in women, and as ex-drinkers if their alcohol consumption was >4 SDU units/day in men and >2.5 SDU/day in women, but they were not currently drinking [59]. For BMI, following Spanish Society for the Study of Obesity criteria, individuals were classified as underweight (BMI < 18.5), healthy weight (18.5 < BMI < 24.9), overweight (25 < BMI < 29.9), class I obese (30 < BMI < 34.9), class II obese (35 < BMI < 39.9) and class III obese (BMI > 40) [60]. The following criteria are based on the guidelines created by the working group of the European Society of Cardiology and the European Society of Atherosclerosis. For dyslipidemia, individuals were classified as dyslipidemic [triglycerides > 150 mg/dL; high-density lipoprotein (HDL) < 40 mg/dL in men and HDL < 48 mg/dL in women; low-density lipoprotein (LDL) > 100 mg/dL; total cholesterol > 200 mg/dL)] and not dyslipidemic. For hypertension, individuals were classified as hypertensive [systolic blood pressure (SBP) > 140 mmHg and diastolic blood pressure (DBP) > 90 mmHg] and non-hypertensive [61]. For diabetes, patients were classified according to American Diabetes Association Criteria into diabetics [steroid diabetes (glucocorticoid-induced hyperglycemia), type I diabetes (fasting blood glucose > 126 mg/dL produced by an autoimmune reaction against insulin-producing cells) and type II diabetes (fasting blood glucose > 126 mg/dL caused by insulin resistance)] and non-diabetics [58]. Both patients with type I and II diabetes have been grouped in the group of cases and controls with diabetes, without excluding any patient. As regards cardiovascular disease, individuals were classified into those with arterial thromboembolism, atrial fibrillation, heart failure, valvular heart disease or venous thromboembolism.

### 4.4. Genetic Variables

#### 4.4.1. DNA Isolation

The DNA samples, isolated from blood or saliva, were obtained from the University Hospital Virgen de las Nieves Biobank, which belongs to the Biobank of the Andalusian Public Health Systems. Blood samples were collected in BD Vacutainer^®^ tubes with EDTA K3 as anticoagulant (3 mL). Saliva samples were collected in 50-mL BD Falcon^TM^ conical tubes (BD, Plymouth, UK). DNA extraction was performed using the DNA QIAamp DNA Mini extraction kit (Qiagen GmbH, Hilden, Germany), in accordance with the specifications provided by the manufacturer, from DNA purification from blood or saliva, and stored at −40°C. DNA concentration and purity were measured using a UV NanoDrop 2000 spectrophotometer^TM^ with the absorbance ratio at 280/260 and 280/230.

#### 4.4.2. Genotyping and Quality Control

The genetic polymorphisms *BsmI* (rs1544410), *TaqI* (rs731236), *ApaI* (rs7975232), *FokI* (rs2228570) and *Cdx2* (rs11568820) were determined by means of real-time polymerase chain reaction (PCR) for allelic discrimination using TaqMan^®^ probes (ABI Applied Biosystems, 7300 Real-Time PCR System), in accordance with the manufacturer’s instructions. The ID assays used for the polymorphisms were as follows: C___2404008_10 for *TaqI* (rs731236), C__28977635_10 for *ApaI* (rs7975232), C__12060045_20 for *FokI* (rs2228570) and C___2880808_10 for *Cdx2* (rs11568820). The *BsmI* polymorphism (rs1544410) was analyzed using an assay customized by ThermoFisher Scientific (Waltham, MA, USA) coded as AN324M4. The criteria for SNPs quality control were: (1) missing genotype rate per SNP < 0.05; (2) minor allele frequency > 0.01; (3) *p* value > 0.05 in Hardy-Weinberg equilibrium test; (4) missing genotype rate between cases and control < 0.05.

#### 4.4.3. Statistical Analysis

Cases and controls were matched by age and gender with 1:1 propensity score matching method. Quantitative data were expressed as the results (±standard deviation) for variables with normal distribution and medians or percentiles (25 and 75) for variables with non-normal distribution. The Shapiro-Wilks test was used to verify normality.

Hardy-Weinberg equilibrium and pairwise haplotype frequencies were estimated, and Lewontin’s D prime (D′) and the linkage disequilibrium coefficient (r2) were calculated. The bivariate association analysis between CVD risk and polymorphisms was performed with multiple models (genotypic, additive, allelic, dominant, and recessive) using Pearson’s Chi-square test and Fisher’s exact test, providing Odds ratio (OR) values and the corresponding 95% confidence interval (95% CI). The models were defined as follows: allelic (D vs. d), dominant ((DD, Dd) vs. dd), recessive (DD vs. (Dd, dd), genotypic (DD vs. dd, and Dd vs. dd) and additive, with D being the minor allele and d, the major allele. The Bonferroni correction was used for multiple comparisons. Unconditional multiple logistic regression models (genotypic, dominant, and recessive) were considered to determine the influence of possible confounding variables on CVD risk. All tests were two-tailed with a significant level of *p* < 0.05 and were performed using free access software for the PLINK whole genome association analysis toolset and the statistical program R 3.2.2 [62,63]. Linkage disequilibrium was performed with Haploview 4.2 software and haplotype analysis with SNPStats, a web tool for the analysis of association studies [64,65].

## 5. Conclusions

The *VDR FokI* polymorphism (rs2228570) was significantly associated with the development of CVD. According to it, this SNP could be used as a risk biomarker for the disease mentioned. Otherwise, no influence was found of the polymorphisms *VDR BsmI* (rs1544410), *TaqI* (rs731236), *ApaI* (rs7975232) and *Cdx2* (rs11568820) on the risk of developing CVD in our patients. Further studies should be done in order to understand and to confirm the role of *VDR* polymorphisms in the CVD.

## Figures and Tables

**Table 1:** Clinico-pathologic characteristics of cardiology cases and controls.

	Cases		Controls		χ²	*p*-Value	Reference	OR	95% CI
	N	*n* (%)	N	*n* (%)					
Gender	246		246						
Male		114 (46.3)		104 (42.3)	0.8237	0.3641			
Female		132 (53.7)		142 (57.7)					
Age	246	70 (63.25, 76)	246	69.5 (63, 76)					
Tobacco consumption	233		203						
Current-smokers		41 (17.6)		27 (13.3)	1.9502	0.3772			
Former-smokers		70 (30.0)		70 (34.5)					
Non-smokers		122 (52.4)		106 (52.2)					
Alcohol consumption	220		173						
Current-drinkers		46 (20.9)		27 (15.6)	2.0823	0.3531			
Former-drinkers		8 (3.6)		5 (2.9)					
Non-drinkers		166 (75.5)		141 (81.5)					
Body Mass Index	182	29.63 ± 5.14	114	28.03 ± 4.89					
Underweight		1 (0.5)		1 (0.9)		0.0066 ^a^	Normal (healthy weight)	1.21	0.05–31.44
Normal (healthy weight)		33 (18.1)		40 (35.1)				1	
Overweight		68 (37.4)		30 (26.3)				2.74	1.47–5.20
Obese Class I (Moderately obese)		46 (25.3)		32 (28.1)				1.74	0.92–3.34
Obese Class II (Severely obese)		29 (15.9)		10 (8.8)				3.51	1.53–8.57
Obese Class III (Very severely obese)		5 (2.7)		1 (0.9)				6.06	0.92–119.19
Dyslipidemia	246		246						
No		158 (64.2)		155 (63.0)	0.0790	0.7786			
Yes		88 (35.8)		91 (37.0)					
Hypertension	246		246						
No		100 (40.6)		115 (46.8)	1.8588	0.1728			
Yes		146 (48)		131 (53.2)					
Diabetes	246		246						
No		170 (69.1)		201 (81.7)	10.5320	0.0012 ^a^	No	1	
Yes		76 (30.9)		45 (18.3)				1.99	1.32–3.06
Disease	246								
Cardiac Arrhythmias		84 (34.15)							
Cardiomyopathy		15 (6.10)							
Cerebrovascular disease		15 (6.10)							
Heart failure		27 (10.98)							
Heart valve disease		46 (18.70)							
Peripheral vascular disease		59 (23.98)							

Shade means the value is significant. ^a^ *p*-value for *t* test.

**Table 2:** Influence of *VDR FokI* (rs2228570) gene polymorphism on risk of cardiovascular disease.

Models	Genotype	Cases [*n* (%)]	Controls [*n* (%)]	*p*-Value ^a^	Adjusted *p*-Value ^b^	OR ^c^	95% CI
Genotypic	TT	45 (18.3)	21 (8.5)	0.0061	0.0305	2.35	1.33–4.28
	CT	99 (40.2)	113 (45.9)			0.96	0.66–1.41
	CC	102 (41.5)	112 (45.5)			1	
Dominant	T	144 (58.5)	134 (54.5)	0.3631	1		
	CC	102 (41.5)	112 (45.5)				
Recessive	TT	45 (18.3)	21 (8.5)	0.0022	0.0075	2.39	1.39–4.24
	C	201 (81.7)	225 (91.5)				
Allelic	T	189 (38.41)	155 (31.50)	0.0230	0.1151		
	C	303 (61.59)	337 (68.50)				
Additive	-	-	-	0.02673 ^d^	0.1336		

CI: confidence interval; OR: odds ratio. ^a^ *p*-value for χ^2^-test. ^b^ *p*-value for Bonferroni correction. ^c^ Unadjusted or crude ORs.^d^ *p*-value for *t* test. Shade means the value is significant.

**Table 3:** Influence of clinical characteristic and *VDR FokI* (rs2228570) gene polymorphism on risk of cardiovascular disease.

	Genotypic						Dominant			Recessive			Additive		
	TT vs. CC			CT vs. CC			T vs. CC			TT vs. C			T vs. C		
	*p*-Value	OR	95% CI	*p*-Value	OR	95% CI	*p*-Value	OR	95% CI	*p*-Value	OR	95% CI	*p*-Value	OR	95% CI
Body Mass Index	0.0265	1.06	1.01–1.12	0.0265	1.06	1.01–1.12	0.0458	1.05	1.00–1.11	0.0413	1.05	1.00–1.11	0.0634	1.05	0.99–1.10
Diabetes															
Yes	0.0359	1.91	1.06–3.55	0.0359	1.91	1.06–3.55	0.0468	1.83	1.02–3.38	0.0316	1.94	1.07–3.59	0.0372	1.89	1.05–3.50
*VDR FokI* (rs2228570)	0.0430	2.30	1.06–5.37	0.2286	0.72	0.43–1.22	0.8648	0.95	0.58–1.57	0.0099	2.71	1.31–6.07	0.2151	1.24	0.88–1.76

Shade means the value is significant.

**Table 4:** Haplotype association with response.

	Rs1544410	Rs7975232	Rs731236	Freq	OR (95% CI)	*p*-Value
1	G	C	T	0.4613	1.00	---
2	A	A	C	0.3685	0.92 (0.70–1.22)	0.570
3	G	A	T	0.1079	0.81 (0.52–1.24)	0.330
4	A	A	T	0.0251	1.11 (0.51–2.38)	0.800
5	G	A	C	0.0189	0.34 (0.12–0.98)	0.047
6	A	C	T	0.0114	1.22 (0.41–3.65)	0.730
Rare	*	*	*	0.0069	0.54 (0.12–2.49)	0.430

Freq: haplotype frequency. Shade means the value is significant. * reference to rare haplotypes as there is no symbol to identify the group.

## Supplementary Materials

The following are available online at https://www.mdpi.com/article/10.3390/ijms23158686/s1.
