# Supplementary material for: Vitamin D-Related Single Nucleotide Polymorphisms as Risk Biomarker of Cardiovascular Disease
Source: Int J Mol Sci. 2022 Aug 4;23(15):8686. doi: 10.3390/ijms23158686 (PMC9368814; doi:10.3390/ijms23158686)
Supplement: Supplementary file 1 [file ijms-23-08686-s001.zip › Table S1.pdf]

Table S1. Hardy-Weinberg Equilibrium

Chr	SNP	Sample	Minor Allele	Major Allele	Genotype counts	Observed heterozygosity	Expected heterozygosity	p-value
12	rs1544410	ALL	A	G	85/233/174	0.4736	0.4836	0.6419
12	rs1544410	AFF	A	G	43/116/87	0.4715	0.484	0.6936
12	rs1544410	UNAFF	A	G	42/117/87	0.4756	0.4833	0.7930
12	rs11568820	ALL	A	G	30/185/277	0.376	0.374	1
12	rs11568820	AFF	A	G	13/96/137	0.3902	0.373	0.6075
12	rs11568820	UNAFF	A	G	17/89/140	0.3618	0.375	0.6098
12	rs2228570	ALL	T	C	66/212/214	0.4309	0.4548	0.2360
12	rs2228570	AFF	T	C	45/99/102	0.4024	0.4732	0.0217
12	rs2228570	UNAFF	T	C	21/113/112	0.4593	0.4316	0.3759
12	rs7975232	ALL	C	A	118/236/138	0.4797	0.4992	0.4161
12	rs7975232	AFF	C	A	56/116/74	0.4715	0.4973	0.4420
12	rs7975232	UNAFF	C	A	62/120/64	0.4878	0.5	0.7032
12	rs731236	ALL	C	T	77/234/181	0.4756	0.4777	0.9249
12	rs731236	AFF	C	T	43/115/88	0.4675	0.4833	0.5996
12	rs731236	UNAFF	C	T	34/119/93	0.4837	0.4712	0.7866
Chr: Chromosome								
