# Supplementary material for: Vitamin D-Related Single Nucleotide Polymorphisms as Risk Biomarker of Cardiovascular Disease
Source: Int J Mol Sci. 2022 Aug 4;23(15):8686. doi: 10.3390/ijms23158686 (PMC9368814; doi:10.3390/ijms23158686)
Supplement: Supplementary file 1 [file ijms-23-08686-s001.zip › Table S2.pdf]

Table S2. Linkage disequilibrium

Chr	BP	SNP	Chr	BP	SNP	R2	D’
12	47846052	rs1544410	12	47845054	rs7975232	0.5439	0.9175
12	47846052	rs1544410	12	47844974	rs731236	0.7131	0.9093
12	47845054	rs7975232	12	47844974	rs731236	0.5697	0.9630
Chr: Chromosome; BP: Physical position (base-pair)							
