# Supplementary material for: Vitamin D-Related Single Nucleotide Polymorphisms as Risk Biomarker of Cardiovascular Disease
Source: Int J Mol Sci. 2022 Aug 4;23(15):8686. doi: 10.3390/ijms23158686 (PMC9368814; doi:10.3390/ijms23158686)
Supplement: Supplementary file 1 [file ijms-23-08686-s001.zip › Table S3.pdf]

Table S3. Minor allele frequencies of SNPs.

Chr	SNP	Minor Allele	Major Alelle	MAF
12	rs1544410	A	G	0.4096
12	rs11568820	A	G	0.249
12	rs2228570	T	C	0.3496
12	rs7975232	C	A	0.4797
12	rs731236	C	T	0.3943
Chr: Chromosome; MAF: Minor allele frequency				
