# Supplementary material for: Vitamin D-Related Single Nucleotide Polymorphisms as Risk Biomarker of Cardiovascular Disease
Source: Int J Mol Sci. 2022 Aug 4;23(15):8686. doi: 10.3390/ijms23158686 (PMC9368814; doi:10.3390/ijms23158686)
Supplement: Supplementary file 1 [file ijms-23-08686-s001.zip › Table S4.pdf]

Table S4. Haplotype frequencies estimation.

	rs1544410	rs7975232	rs731236	Total	Control group	Case group	Cumulative frequency
1	G	C	T	0.4613	0.4449	0.4779	0.4613
2	A	A	C	0.3685	0.3698	0.3674	0.8298
3	G	A	T	0.1079	0.1150	0.1009	0.9377
4	A	A	T	0.0251	0.0223	0.0277	0.9628
5	G	A	C	0.0189	0.0295	0.0081	0.9817
6	A	C	T	0.0114	0.0093	0.0134	0.9930
7	A	C	C	0.0046	0.0092	0	0.9977
8	G	C	C	0.0023	0	0.0046	1

Rare haplotypes are coloured as grey.
