# Supplementary material for: Vitamin D-Related Single Nucleotide Polymorphisms as Risk Biomarker of Cardiovascular Disease
Source: Int J Mol Sci. 2022 Aug 4;23(15):8686. doi: 10.3390/ijms23158686 (PMC9368814; doi:10.3390/ijms23158686)
Supplement: Supplementary file 1 [file ijms-23-08686-s001.zip › Table S5.pdf]

Table S5. Polymorphisms and association with risk of cardiovascular disease.

SNP	Minor Allele	Mayor Allele	Model	Cases	Controls	χ²	p-value χ²	p-value Fisher
rs1544410	A	G	Genotypic	43/116/87	42/117/87	0.0161	0.9920	1
			Additive	202/290	201/291	0.0041	0.9488	0.9488
			Allelic	202/290	201/291	0.0042	0.9483	1
			Dominant	159/87	159/87	0	1	1
			Recessive	43/203	42/204	0.0142	0.9051	1
rs11568820	A	G	Genotypic	13/96/137	17/89/140	0.8307	0.6601	0.6505
			Additive	122/370	123/369	0.0055	0.9411	0.9411
			Allelic	122/370	123/369	0.0054	0.9412	1
			Dominant	109/137	106/140	0.0744	0.7851	0.8558
			Recessive	13/233	17/229	0.5680	0.4511	0.5726
rs2228570	T	C	Genotypic	45/99/102	21/113/112	10.12	0.0063	0.0061
			Additive	189/303	155/337	4.909	0.0267	0.0267
			Allelic	189/303	155/337	5.167	0.0230	0.0273
			Dominant	144/102	134/112	0.827	0.3631	0.4131
			Recessive	45/201	21/225	10.08	0.0015	0.0022
rs7975232	C	A	Genotypic	56/116/74	62/120/64	1.098	0.5777	0.5923
			Additive	228/264	244/248	1.003	0.3165	0.3165
			Allelic	228/264	244/248	1.042	0.3073	0.3385
			Dominant	172/74	182/64	1.007	0.3156	0.3665
			Recessive	56/190	62/184	0.4013	0.5264	0.5977
rs731236	C	T	Genotypic	43/115/88	34/119/93	1.258	0.5330	0.5366
			Additive	201/291	187/305	0.8305	0.3621	0.3621
			Allelic	201/291	187/305	0.834	0.3611	0.3964
			Dominant	158/88	153/93	0.2185	0.6402	0.7085
			Recessive	43/203	34/212	1.247	0.2641	0.3209
Chr: chromosome; NA: not applicable								
